# A rare case with fetal autoimmune heart block and *KNCH2* variant–induced long QT syndrome: a controversial opinion on prenatal management strategy

**DOI:** 10.1186/s12872-023-03198-8

**Published:** 2023-03-27

**Authors:** Li Wei, Jiahao Wu, Peihuan Xie, Xiaoliang Liu, Yimin Hua, Kaiyu Zhou, Chuan Wang, Yifei Li

**Affiliations:** Key Laboratory of Birth Defects and Related Diseases of Women and Children of MOE, Department of Pediatrics, West China Second University Hospital, Sichuan University, Section Renmin S. Rd, 20 3Rd, Chengdu, 610041 Sichuan China

**Keywords:** Fetal autoimmune AVB, Long QT syndrome, KCNH2, Prenatal management, Dexamethasone, Case report

## Abstract

**Background:**

Among all fetal heart block patients, > 50% cases are associated with maternal autoimmune diseases, and such patients should receive treatment. However, nearly half of fetal heart block cases involve a mother with negative results following autoimmune antibody screening. A few studies have reported long QT syndrome (LQTS) can also present as a severe fetal bradycardia, which does not respond to fetal treatment. Herein, we reported a rare case of an infant who presented with high-degree autoimmune-mediated fetal atrioventricular block (AVB) with LQTS induced by a novel *KCNH2* variant. This case led us to review our prenatal therapeutic strategy.

**Case presentation:**

A 1-year-old boy presented to our heart center having experienced syncope 5 times in the past year. He had previously presented with fetal bradycardia during the fetal stage from 27 + 3 gestational weeks. The fetal echocardiography demonstrated AVB (2:1 transmission). As the maternal autoimmune antibody results were positive, his mother had received dexamethasone treatment during pregnancy; subsequently, the fetal AVB had changed from 2:1 to 4:3 transmission with elevated ventricular beating rates. However, this patient was identified to have complete AVB after birth. The initial electrocardiogram and Holter measurements at hospital administration showed complete AVB, pleomorphic ventricular tachycardia, a prolonged QT interval (QT = 602 ms, corrected QT = 538 ms), and wide and deep inverted T-waves. Meanwhile, torsades de pointes could be observed in several transit ventricular tachycardias based on Holter monitoring review. Genetic testing revealed *KCNH2* c.2483G > A variant–induced LQTS. An implantable cardioverter defibrillator device and permanent pacemaker were both considered as therapeutic alternations; his parents ultimately accepted the implantation of a permanent pacemaker.

**Conclusions:**

For fetuses with autoimmune-mediated AVB, intrauterine treatment should still be pursued immediately. However, once the treatment outcomes are deemed unacceptable or unexpected, other genetic variant–related channelopathies should be highly suspected. If the fetus lacks a positive family history, fetal genetic testing should be recommended to improve the prognosis of such patients by introducing integrative therapeutic strategies between the prenatal and postnatal phases.

## Introduction

Fetal heart block is a kind of rare and threatening disease with an incidence of 1/20000–1/15000 in all pregnancies across the world. It is considered to be the most commonly observed type of fetal bradycardia. Among all fetal heart block patients, > 50% cases are associated with maternal autoimmune diseases, and the incidence of fetal heart block in pregnancies with positive anti–SS-A(Ro) and anti–SS-B(La) test results is 2%–5% [1]. Further, an estimated 12%–25% of newborns suffer neonatal lupus in a subsequent pregnancy. Such autoimmune antibodies include anti-nuclear antibodies (ANAs), which can move from the maternal circulation to the fetal side through the placenta via FcγRn and induce inflammation activities. In the fetal heart, Ro52, Ro60, and La antigens are located in the nuclei of cardiomyocytes and cardiac conduction cells. Ro52 is involved in the regulation of interferon regulatory factor–mediated immune responses. This inflammation activity induces immune attacks and fibrotic responses, resulting in heart conductive cell damage, leading to the occurrence of fetal heart block. As such, in autoimmune antibody–positive pregnancies, intravenous immunoglobulin (IVIG) and dexamethasone (DEX) are typically administered to reverse the fetal heart block. However, current therapeutic strategies have shown several disadvantages, only a small proportion of fetuses with heart block could be returned into normal sinus rhythm with 1:1 AV conduction. While a relatively high mortality rate has been observed even after treatment.

Moreover, nearly half of fetal heart block cases involve a mother with negative autoimmune antibody screening results. To identify the causes of fetal heart block and develop a subsequent treatment strategy for such patients are involved greatly challenges. A few studies have reported that long QT syndrome (LQTS) could also present as severe fetal bradycardia [2]. As LQTS, a kind of inherit genetic disorder, was not induced by autoimmune attacks, it was definitely irresponsible for intrauterine IVIG and DEX treatment, it is difficult to distinguish between them during pregnancy unless fetal genetic testing is performed to identify genetic variant–related arrhythmias [3]. Moreover, cases in which the autoimmune antibodies were negative are highly associated with adverse arrhythmias postnatally, including LQTS and complete atrioventricular block (CAVB). It was reported that 5%–30% of CAVB patients can develop torsades de pointes (TDP) as well as LQTS [4]. CAVB and LQTS carry a high risk of evolving into sharp-angle TDP, which leads to sudden cardiac death (SCD); this trend may arise from the downregulation of the potassium channel current and a prolonged QT interval [5].

Herein, we report the rare case of an infant who presented with high-degree autoimmune-mediated fetal AVB. In utero, the combination of IVIG and DEX as treatment could only partially elevate his ventricular beating rate. After birth, the patient suffered several instances of recurrent syncope, and genetic testing confirmed his condition was the result of de novo* KCNH2* variant–induced LQTS. This case led us to review our prenatal therapeutic strategy for such patients and examine the postnatal therapy plan for this patient who presented with TDP and an extremely high possibility of SCD. We would also like to mention the importance of prenatal genetic screening even among pregnancies with positive autoimmune antibodies.

## Case presentation

### Ethical compliance

This report was approved by the ethics committee of the West China Second Hospital of Sichuan University (approval number 2014–034). Informed consent was obtained from the patient’s parents prior to performing whole-exon sequencing and for the inclusion of the patient’s clinical and imaging details in subsequent publications.

### History of illness

**Fig. 1:**
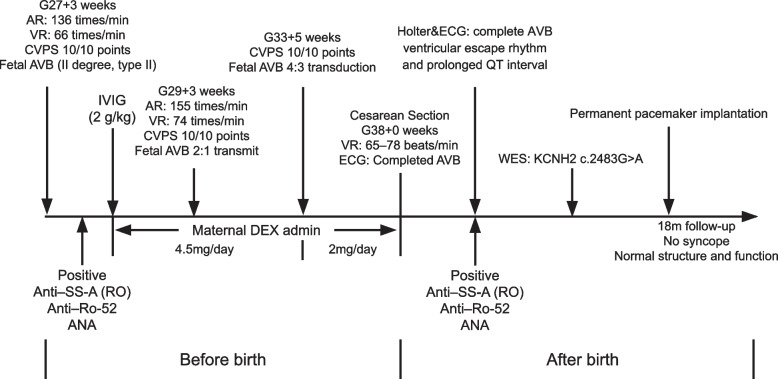
Timeline for clinical management of the proband. AR, atrial beating rate; AVB, atrioventricular block; DEX, dexamethasone; IVIG, intravenous immune globulin; VR ventricular beating rate

A 1-year-old boy presented to our heart center having experienced syncope 5 times in the past year, with timely cardiopulmonary resuscitation performed all times. After the last time of syncope, the patient was transferred to our cardiac intensive care unit within 30 min. A detailed history of illness was available. This boy had presented with fetal bradycardia during the fetal stage from 27 + 3 gestational weeks, and fetal echocardiography demonstrated AVB (2:1 transmission). As the maternal autoimmune antibody result was positive, his mother had received DEX during pregnancy. Subsequently, the fetal AVB had changed from 2:1 to 4:3 transmission with elevated ventricular beating rates, which suggested a therapeutic benefit of DEX. However, this patient was confirmed to have complete AVB after birth with a lower ventricular beating rate of 65–95 beats/min. As his SS-A (Ro), Ro-53, and ANA tests were positive, he was treated with IVIG (1 g/kg) in our neonatal department. However, there was no recovery of the heart transduction disorder. At the time, his parents declined the implantation of an implantable cardioverter-defibrillator and pacemaker, and the patient was discharged without any further treatment. His parents also had no positive or related family history of arrhythmia, cardiomyopathy, congenital heart disease, or coronary artery disease. The clinical manifestation has been summarized in Fig. 1 as a timeline.

### Physical, laboratory, and imaging examinations and treatment

**Fig. 2:**
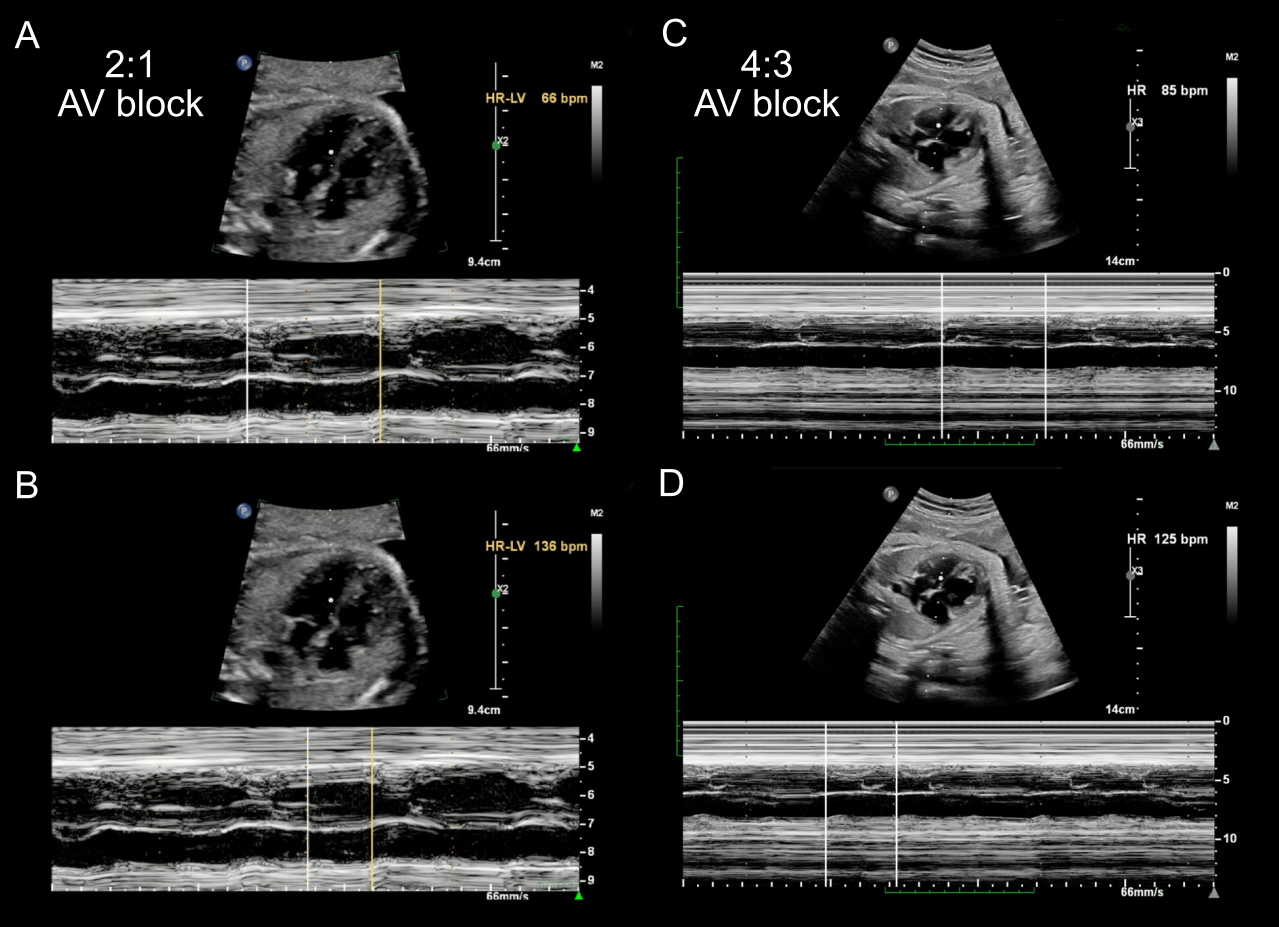
Fetal echocardiography to determine bradycardia. **A** M-mode echocardiography demonstrated the ventricular rate of 66 beats per minute before treatment. **B** M-mode echocardiography demonstrated the atrial rate of 136 beats per minute before treatment, indicating a 2:1 AV block. **C** M-mode echocardiography demonstrated the ventricular rate of 85 beats per minute after treatment. **B** M-mode echocardiography demonstrated the atrial rate of 125 beats per minute after treatment, indicating a 4:3 AV block

The first fetal echocardiography exam was performed at 27 + 3 gestational weeks during the fetal stage. The development of 4 chambers and valvar movements was normal. The atrial beating rate was 136 times/min, while the ventricular beating rate was 66 times/min (Fig. 2A and B), and the cardiovascular system profile was normal (10/10 points). As such, a diagnosis of fetal AVB (II degree, type II) was identified. The maternal autoimmune antibody test results were positive for anti–SS-A (RO) (> 400.0 RU/mL, n.v. < 20 RU/mL), anti–Ro-52 (> 400.0 RU/mL, n.v. < 20 RU/mL), and ANAs (1:1000, n.v. < 1:100). The immunoglobulin M result for coxsackievirus was also positive. After that, IVIG (2 g/kg in total) and DEX (4.5 mg/day) were provided to the mother. During fetal follow-up, at 29 + 3 gestational weeks, a fetal AVB of 2:1 transduction was still observed, as the atrial beating rate was 144–155 beats/min and the ventricular beating rate was 65–74 times/min. However, at 33 + 5 gestational weeks, some recovery was identified as fetal echocardiography revealed a change in the AVB from 2:1 to 4:3 transduction, and the cardiovascular system profile remained normal. As such, it seemed that treatment with IVIG and DEX had had some benefits and therapeutic effects on the heart transduction.

**Fig. 3:**
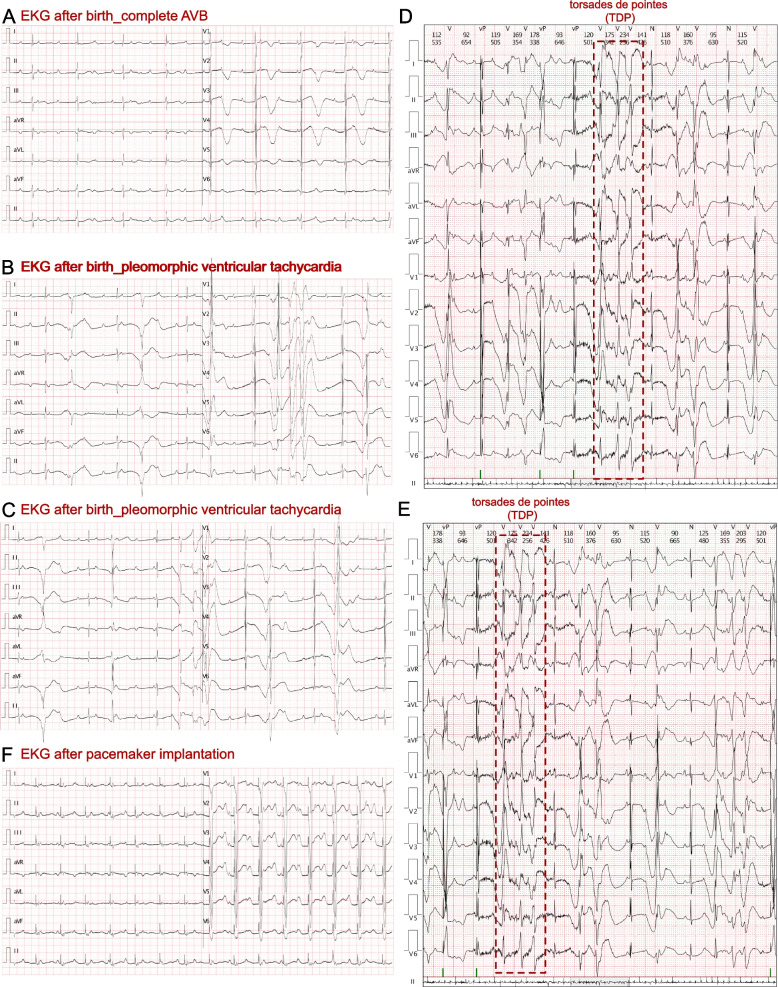
Clinical manifestations in EKG. **A** EKG revealed complete AVB after birth. **B** and **C** Pleomorphic ventricular tachycardia had been identified. **D** and **E** Holter demonstrated several times of short torsades de pointes (TDP). **F** EKG after pacemaker implantation

Cesarean section was performed at 38 gestational weeks. After birth, a bradycardia was also documented, and the ventricular beating rate was 65–78 beats/min, with an acceptable SpO_2_ of around 98%. An electrocardiogram (ECG) of the boy taken during the neonatal stage demonstrated complete AVB (Fig. 3A). Moreover, Holter monitoring revealed an escape rhythm with complete AVB, and the average ventricular beating rate was 69 beats/min. In total, 8867 times (9.9% of total beats) of pleomorphic premature ventricular contractions were recorded, and a significantly prolonged QT interval (550–610 ms) was noted. Besides, the autoimmune antibody test results of this neonate were positive for anti–SS-A (RO) (283.1 RU/mL, n.v. < 20 RU/mL), anti–Ro-52 (> 400.0 RU/mL, n.v. < 20 RU/mL), and ANAs (1:1000, n.v. < 1:100). Echocardiography confirmed the presence of patent ductus arteriosus, patent foramen ovale, mitral regurgitation (mild to moderate), tricuspid regurgitation (mild) with an ejection fraction of 61%. Cardiac magnetic resonance imaging failed to address any abnormal signaling in myocardium. The patient then received IVIG administration for 2 days (400 mg/kg/day), but his parents refused further treatment. There was no change in the ECG. However, a diagnosis of LQTS was suspected for this patient.

At this time of admission, the initial ECG and Holter measurements at hospital administration suggested complete AVB, pleomorphic ventricular tachycardia, a prolonged QT interval (QT = 602 ms, corrected QT = 538 ms), and wide and deep inverted T-waves (Fig. 3B and C). Interestingly, TDP could be observed in several transit ventricular tachycardias based on Holter monitoring (Fig. 2D and E). In addition, the serum levels of cardiac troponin I (cTnI) (n.v. < 0.06 µg/L) and B-type natriuretic peptide (n.v. < 100 pg/mL) were normal. Fulminant myocarditis was also ruled out based on negative results for all tests for suspected virus and myocardial injury marker changes. Echocardiography demonstrated a normal cardiac structure with patent ductus arteriosus, mitral regurgitation (moderate), and tricuspid regurgitation (mild) without myocardial hypertrophy, pathological ventricular dilatation, left ventricular diastolic dysfunction. Therefore, there was no convincing evidence for a diagnosis of cardiomyopathy. As LQTS had been highly suspected, whole-exon sequencing was performed to identify any potential genetic variants.

### Molecular results

**Fig. 4:**
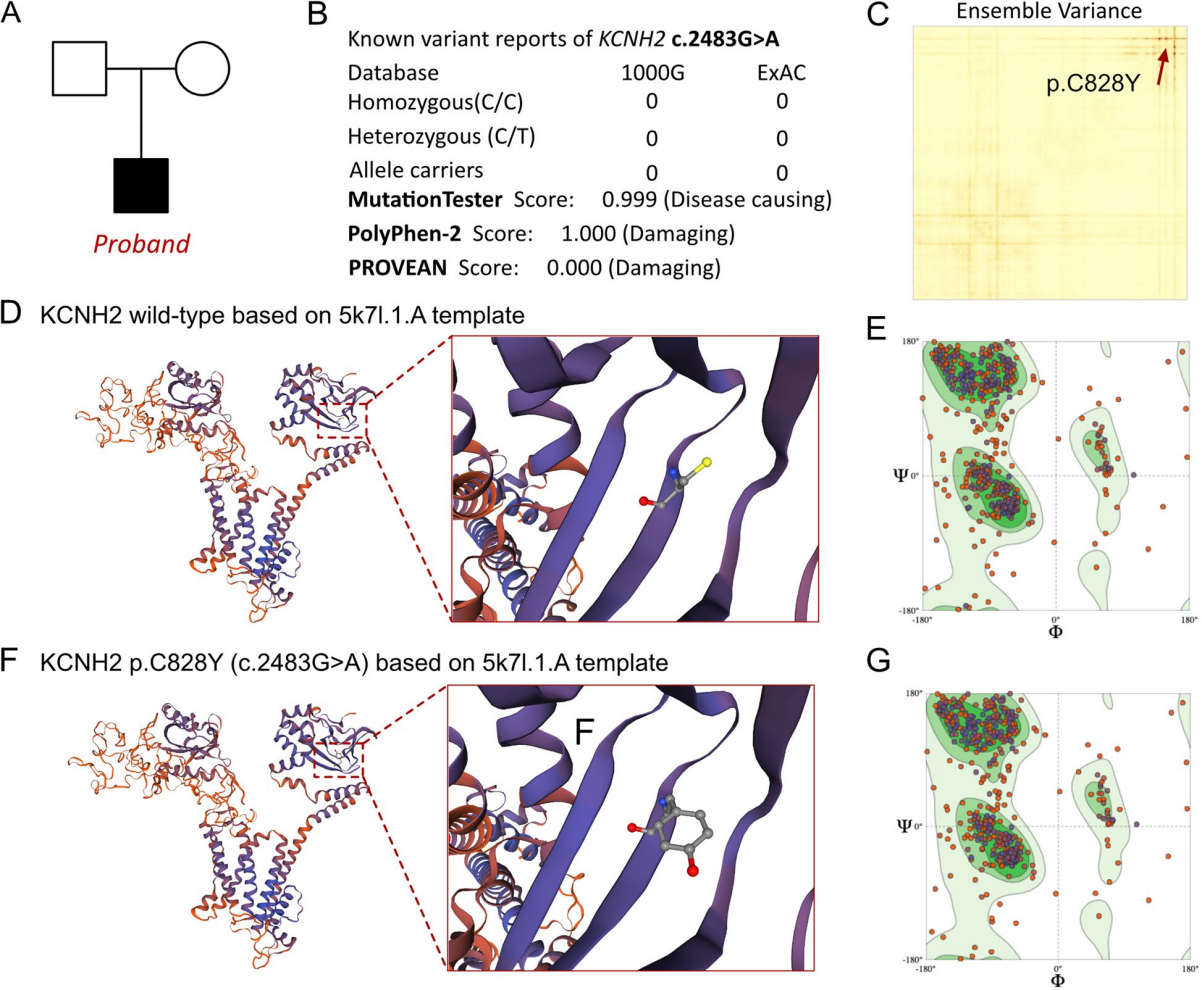
Effects of *KCNH2* c.2483G > A mutation on molecular protein structure. **A** Family pedigree revealed the proband carrying a de novo* KCNH2* variant. **B** Summary of current reports on the individuals of *KCNH2* and its predicted damages on molecular function of c.2843G > A variant. **C** Ensemble variance between wild type and mutant protein structure. **D** and **F** SWISS-MODEL to predict the variant’s wild-type and mutated protein crystal structures using 5k7l.1.A template, and structural changes identified. **E** Ramachandran plots of wild-type KCNH2. G. Ramachandran plots of KCNH2 with p.L908P variant. **D** SWISS-MODEL to predict the variant’s wild-type and mutated protein crystal structures using 5tby.1.A template, and structural changes identified

Based on laboratory analyses and the patient’s clinical manifestations, a genetic disorder was strongly suspected. To evaluate any potential genetic causes of this patient’s condition, a peripheral blood sample was obtained from the patient for genetic sequencing analysis. Whole-exon sequencing was performed using the Illumina NovaSeq 6000 platform, and a de novo c.2483G > A (p.C828Y) heterozygous mutation was identified in the *KCNH2* gene. Neither of the patient’s parents carried this variant (Fig. 4A). According to the American College of Medical Genetics, these variants have an uncertain pathogenicity (PM2_Supporting + PP3 + PP2 + PP4). The variant we identified, *KCNH2* c.2483G > A, had not been reported in any population; this is the first report of this variant (Fig. 4B). An analysis performed with MutationTaster revealed that this mutation is considered pathogenic due to amino acid sequence changes, the protein features affected, and a loss of helix superstructure (probability = 0.999 for c.2483G > A). PolyPhen 2.0 predicted that this mutation of p.C828Y to be “probably damaging” (score = 1.0, sensitivity = 0.00, specificity = 1.00). The SWISS-MODEL tool was used to analyze stability after amino acid changes (Fig. 4C). Ramachandran plots indicated that amino acid positions were altered (Fig. 4E and G). Rebuilding the molecular structure based on a 5k7l.1.A templet resulted in the identification of residue changes between Cys and Tyr at 828 (Fig. 4D and F). Using SWISS-MODEL protein stability prediction tools, ensemble changes among all the coded amino acids exhibited significant variance (Fig. 4C). Three types of calculation methods all demonstrated significant destabilizing changes (mCSM =  − 1.137 kcal/mol; DUET =  − 1.235 kcal/mol; SDM =  − 1.420 kcal/mol).

### Outcome and follow-up

After the patient was transferred to the cardiac intensive care unit, a temporary pacemaker had been implanted for positive treatment. After we obtained the genetic result of *KCNH2* c.2483G > A, we considered the patient to remain at extremely high risk of SCD given that TDP had been documented by Holter monitoring. As such, an implantable cardioverter-defibrillator and a permanent pacemaker were each considered as therapeutic alternations. Ultimately, his parents accepted the implantation of a permanent pacemaker. An ECG taken after permanent pacemaker implantation demonstrated a ventricular beating rate that remained at 90 beats/min and a QT interval of 514 ms (corrected QT = 642 ms) (Fig. 2G). During 18 months of follow-up, the patient did not suffer syncope again. Furthermore, echocardiography and cardiac magnetic resonance imaging demonstrated a normal heart structure and function.

## Discussion and conclusions

Newborns with inherited arrhythmias have a higher risk of congenital heart disease or heart failure [6]. When a patient presents with intrauterine manifestations of fetal AVB and ventricular tachycardia, congenital LQTS should be highly suspected, and its strong correlation with the clinical course of malignancy should be specially evaluated [7]. Generally, prolongation of the QT interval is responsible for bradycardia. Once AVB induced by prolongation of the QT interval occurs, it carries a high risk of TDP, which is closely associated with SCD [8]. The occurrence of AVB in concert with LQTS in patients is not due to the disease of the conduction system itself but rather because the ventricular repolarization time is too long and the atrium is activated before the ventricular repolarization is complete [9]. Some patients with TDP induced by AVB with 4 heterozygous missense mutations (28.6%), including *KCNQ1* and *KCNH2,* which share similar molecular functions and result in congenital LQTS, were reported [10]. Furthermore, a 6-year-old boy was diagnosed with CAVB at the age of 5 months with the identification of TDP. However, this patient presented with QT-interval prolongation at the age of 1 year, and he was treated with propranolol (2 mg/kg/day) and pacemaker implantation to avoid SCD attacks. In the report of a series of 287 patients with LQTS from Garson et al., there were 15 patients (5%) with AVB, 13 patients with 2:1 block, and only 2 (0.7%) patients with CAVB [11]. Early awareness of LQTS is critical to prevent adverse clinical outcomes. However, during the fetal stage, LQTS might present as isolated mild sinus bradycardia or 2:1 AVB. As fetal echocardiography is the dominant method to evaluate the cardiac rhythm, we could not exactly discern the electrophysiological activities in utero. As such, LQTS typically fails to be diagnosed during the fetal phase. Furthermore, guidelines also state to use genetic testing to reach a solid molecular diagnosis for such channelopathies after birth. On the other hand, patients with channelopathy-induced fetal bradycardia are not recommended to receive treatment during pregnancy, and structural cardiac defects and cardiomyopathies should be excluded. Only TDP and ventricular tachycardia intrauterine are required to be terminated.

Aside from LQTS, autoimmune-mediated AVB also contributes to a large proportion of fetal bradycardia cases. It was reported that high-titer anti–SS-B/La antibodies, particularly when accompanied by high-titer anti–SS-A/Ro antibodies, can lead to CAVB, sinoatrial node dysfunction, ventricular and junctional tachycardias, and long QT intervals [12]. Accordingly, treatment for autoimmune-mediated fetal AVB is recommended during the fetal stage by way of DEX and IVIG maternal administration, which has had efficient benefits for fetal prognosis. However, there are some situations where the use of such therapeutic strategies leads to complicated issues. Mizuno et al. reported an infant case of CAVB with TDP and no genetic evidence on LQTS of any variants in *KCNQ1*, *KCNH2*, or *SCN5A* [13]. In a study by Michaelsson et al., 7% of patients were identified to have prolonged QT intervals (corrected QT interval > 450 ms) [14]. Two mechanisms were proposed for the development of QT prolongation in patients with congenital AVB. First, a conduction disorder and bradycardia may be the initial phenomena, followed by the development of repolarization changes. Alternatively, patients with congenital AVB might present with the phenotype of recessive congenital LQTS [15]. It had been reported that congenital AVB could combine with LQTS [16]. Escher et al. determined that 59 patients (21% of patients) had LQTS among 273 patients with congenital CAVB [14]. In AVB patients, TDP might be a sign of potential genetic susceptibility to reduced repolarization reserve and a phenotypic manifestation of potential congenital LQTS [17, 18]. In other words, LQTS may affect a great portion of patients with congenital AVB, and those with LQTS receive few benefits from fetal treatment compared to other types of congenital AVB.

Accordingly, in this case, genetic results revealed a rare presentation of *KCNH2*-positive LQTS combined with autoimmune-mediated congenital high-degree AVB. The maternal tests for autoimmune antibodies were positive, which means that this fetus should have been treated for AVB. At the beginning, the fetus presented with 2:1 AVB, which evolved into 4:3 AVB after 4 weeks of treatment; however, treatment was continued through delivery. As such, this case led us to consider the use of fetal genetic testing for channelopathies, even in patients with positive maternal autoimmune antibody results. The long-term administration of DEX in late gestation can result in several developmental disorder for fetuses. It was hard to balance the benefits and risks of long-term fetal intrauterine DEX exposure in this case. As LQTS could presented as 2:1 AV conduction in fetal stage, which was irresponsible for IVIG and steroid therapy. It recommend fetal genetic sequencing analysis would be much helpful in managing the treatment irresponsible fetuses. At that time, a fetal genetic diagnosis of LQTS would have been sufficient.

In summary, for fetuses with autoimmune-mediated AVB, intrauterine treatment should still be pursued immediately. However, once the treatment outcomes are deemed unacceptable or unexpected, other genetic variant–related channelopathies should be highly suspected. If such a fetus lacks a positive family history, fetal genetic testing should be recommended to improve the prognosis of such patients by introducing an integrative therapeutic strategy between the prenatal and postnatal phases.

## Data Availability

Data sets used in this study are available from the corresponding author upon reasonable request.

## Abbreviations

ANAs: Anti-nuclear antibodies
AVB: Atrioventricular block
CAVB: Complete atrioventricular block
DEX: Dexamethasone
ECG: Electrocardiogram
IVIG: Intravenous immunoglobulin
LQTS: Long QT syndrome
TDP: Torsades de pointes
MAFs: Minor allele frequencies
SCD: Sudden cardiac death
SDM: Site-directed mutator
